# Personalised surveillance for serrated polyposis syndrome: results from a prospective 5-year international cohort study

**DOI:** 10.1136/gutjnl-2018-318134

**Published:** 2019-04-13

**Authors:** Arne GC Bleijenberg, Joep EG IJspeert, Yasmijn J van Herwaarden, Sabela Carballal, María Pellisé, Gerhard Jung, Tanya M Bisseling, Iris D Nagetaal, Monique E van Leerdam, Niels van Lelyveld, Xavier Bessa, Francisco Rodríguez-Moranta, Barbara Bastiaansen, Willemijn de Klaver, Liseth Rivero, Manon CW Spaander, Jan Jacob Koornstra, Luis Bujanda, Francesc Balaguer, Evelien Dekker

**Affiliations:** 1 Gastroenterology, Cancer Center Amsterdam, Amsterdam UMC, University of Amsterdam, Amsterdam, The Netherlands; 2 Department of Gastroenterology and Hepatology, Radboud University Medical Center, Nijmegen, The Netherlands; 3 Gastroenterology Department, Hospital Clinic de Barcelona, Barcelona, Spain; 4 Centro de Investigación Biomédica en Red de Enfermedades Hepáticas y Digestivas (CIBEREHD), Institut d’Investigacions Biomèdiques August Pi i Sunyer (IDIBAPS), Barcelona, Spain; 5 Department of Pathology, Radboud University Medical Center, Nijmegen, The Netherlands; 6 Gastroenterology and Hepatology, Netherlands Cancer Institute, Amsterdam, The Netherlands; 7 Gastroenterology and Hepatology, St Antonius Hospital, Nieuwegein, The Netherlands; 8 Gastroenterology, Institut Hospital del Mar d’Investigacions Mediques, Barcelona, Spain; 9 Gastroenterology Department, Bellvitge University Hospital, Barcelona, Spain; 10 Gastroenterology and Hepatology, Erasmus University Medical Center, Rotterdam, The Netherlands; 11 Gastroenterology and Hepatology, University Medical Center Groningen, Groningen, The Netherlands; 12 Gastroenterology, Donostia Hospital, San Sebastian, Spain

**Keywords:** colorectal cancer, colonic polyps, polyposis

## Abstract

**Background and aims:**

Serrated polyposis syndrome (SPS) is associated with an increased risk of colorectal cancer (CRC). International guidelines recommend surveillance intervals of 1–2 years. However, yearly surveillance likely leads to overtreatment for many. We prospectively assessed a surveillance protocol aiming to safely reduce the burden of colonoscopies.

**Methods:**

Between 2013 and 2018, we enrolled SPS patients from nine Dutch and Spanish hospitals. Patients were surveilled using a protocol appointing either a 1-year or 2-year interval after each surveillance colonoscopy, based on polyp burden. Primary endpoint was the 5-year cumulative incidence of CRC and advanced neoplasia (AN) during surveillance.

**Results:**

We followed 271 SPS patients for a median of 3.6 years. During surveillance, two patients developed CRC (cumulative 5-year incidence 1.3%[95% CI 0% to 3.2%]). The 5-year AN incidence was 44% (95% CI 37% to 52%), and was lower for patients with SPS type III (26%) than for patients diagnosed with type I (53%) or type I and III (59%, p<0.001). Most patients were recommended a 2-year interval, and those recommended a 2-year interval were not at increased risk of AN: AN incidence after a 2-year recommendation was 15.6% compared with 24.4% after a 1-year recommendation (OR 0.57, p=0.08).

**Conclusion:**

Risk stratification substantially reduced colonoscopy burden while achieving CRC incidence similar to previous studies. AN incidence is considerable in SPS patients, but extension of surveillance intervals was not associated with increased AN in those identified as low-risk by the protocol. We identified SPS type III patients as low-risk group that might benefit from even less frequent surveillance.

**Trial registration number:**

The study was registered on http://www.trialregister.nl; trial-ID NTR4609.

**Significance of this study**

**What is already known on this subject?**

- Serrated polyposis syndrome (SPS) is associated with a high prevalence of colorectal cancer (CRC), both at baseline as well as during surveillance.
- Endoscopic surveillance is needed to prevent CRC.
- Current surveillance recommendations might be too stringent for many SPS patients, but risk stratification tools are lacking.

**What are the new findings?**

- Extension of surveillance intervals is not associated with an increased incidence of advanced neoplasia.
- A substantial reduction in colonoscopy burden can be achieved by using patient-specific risk factors for determining optimal surveillance intervals.

**How might it impact on clinical practice in the foreseeable future?**

- Our proposed personalised surveillance protocol might help to achieve a substantial reduction in colonoscopy burden for SPS patients.

## Introduction

Serrated polyposis syndrome (SPS) is characterised by the presence of numerous colonic serrated polyps (SPs), and is accompanied by a substantially increased colorectal cancer (CRC) risk.^1–4^ Hence, close endoscopic surveillance is essential to prevent CRC development.^1 2 5^ Although previously considered to be uncommon, recent evidence estimates a prevalence of up to 1:111 (0.9%) individuals in faecal occult blood test-based screening cohorts and up to 1:238 (0.42%) in primary screening cohorts fulfil the diagnostic criteria for SPS diagnosis when subsequent surveillance colonoscopies are taken into account, making SPS the most prevalent polyposis syndrome currently known.^6–8^

Since no genetic mutations have been identified to diagnose SPS, diagnosis is based on clinical criteria defined by the WHO.^5^ These include *(1) at least five SPs proximal to the sigmoid, with two or more of these being ≥10 mm; (2) any number of SPs proximal to the sigmoid in an individual who has a first-degree relative with SPS or (3) ≥20 SPs of any size, distributed throughout the colon.*

The prevalence of CRC in patients with SPS has been estimated to range between 15% and 35%.^1 2 4^ Two small cohorts reported a higher CRC incidence of 54%–70%.^9 10^ Although the majority of CRCs in SPS patients occur prior to, or at the time of SPS diagnosis, there also seems to be an increased risk for CRC during surveillance. In three retrospective and one prospective cohorts, the cumulative 5-year incidence of CRC under endoscopic surveillance ranged between 0% and 7.0%.^1–4 11^ This has led to stringent surveillance recommendations worldwide, most of them recommending either annual colonoscopy or colonoscopy every 1–2 years.^12–15^ For example, the US Multi-Society Task Force on CRC recommends annual surveillance colonoscopies for all SPS patients.^12^ Dutch and Spanish guidelines recommend surveillance every 1–2 years,^13 16^ but in daily practice, clinicians tend to stay on the safe side, with median surveillance intervals in recent cohort studies ranging between 1.1 and 1.3 years.^1–3^ All guidelines mention the scarce evidence to support their stringent recommendations, and many authors have expressed the need for prospective evaluation of these surveillance recommendations.^1–3 12^

The prevalence of SPS combined with the stringent surveillance regimens lead to substantial colonoscopy burden. Annual surveillance seems appropriate for some patients, but extension of surveillance intervals beyond 1 year might be safe for the majority. Unfortunately, current guidelines lack risk stratification and are based on a one-size-fits-all principle.^12–15^ Several recent studies suggest that CRC risk depends on patient-specific risk factors, such as a history of advanced SP, advanced adenomas or smoking history.^1–3^ Ideally, such risk factors would facilitate personalised risk stratification and reduction of colonoscopy burden for patients at low risk of CRC. The importance of such risk stratification has been advocated by several authors recently.^17 18^

Therefore, the aim of the current study was to prospectively evaluate the safety and effectivity of a personalised surveillance protocol that uses individual patient characteristics to re-determine the optimal surveillance interval after each surveillance colonoscopy.

## Methods

### Patients and study design

All patients fulfilling WHO SPS criterion I and/or III^5^ were eligible for inclusion if they underwent endoscopic surveillance after successful clearing of all relevant polyps (see the Clearing phase section) between January 2013 and April 2018. To reflect a representative clinical setting, both SPS patients that already underwent surveillance before enrolment, as well as patients that had not undergone any surveillance before enrolment, could be included. Patients were recruited in three Spanish and six Dutch centres of expertise. Patients with a history of proctocolectomy or subtotal colectomy, with known CRC-related germline mutations (ie, *GREM*, *PTEN*, *BMPR1A*, *SMAD4*, *ENG1*, (bi-allelic) *MutYH* or *APC*) or with inflammatory bowel disease were excluded. Individuals fulfilling only WHO criterion II were excluded from the current study because most experts agree that this criterion is insufficient for diagnosis of SPS, although literature supporting this claim is lacking. By excluding this group, we aimed to prevent potential contamination of our cohort with a subgroup of patients with a different CRC risk. Moreover, this was a prospective international multicentre cohort study. Because the surveillance protocol fell within the surveillance recommendations of Dutch and Spanish guidelines and data were collected as part of routine care, the Institutional Review Board of the Academic Medical Center Amsterdam decided that the study fell beyond the legislation regarding Medical Research Involving Human Subjects Act (Wet Medisch wetenschappelijk Onderzoek met mensen (WMO)). Patients were notified about the protocol verbally. The study was registered on the publicly accessible Dutch Trial Register (http://www.trialregister.nl; trial-ID NTR4609).

### Histopathological evaluation

Tissue specimens were routinely processed by GI pathologists. SPs were classified as hyperplastic polyp, sessile serrated lesion (SSL, also known as sessile serrated adenoma or sessile SP) with or without dysplasia, or traditional serrated adenoma (TSA) based on the WHO classification for SP.^5 19^ Advanced adenomas were defined as adenomas ≥10 mm, with villous structure and/or with high-grade dysplasia (HGD). Advanced SPs were defined as any SP >10 mm and/or with presence of dysplasia.

### Surveillance protocol

**Figure 1:**
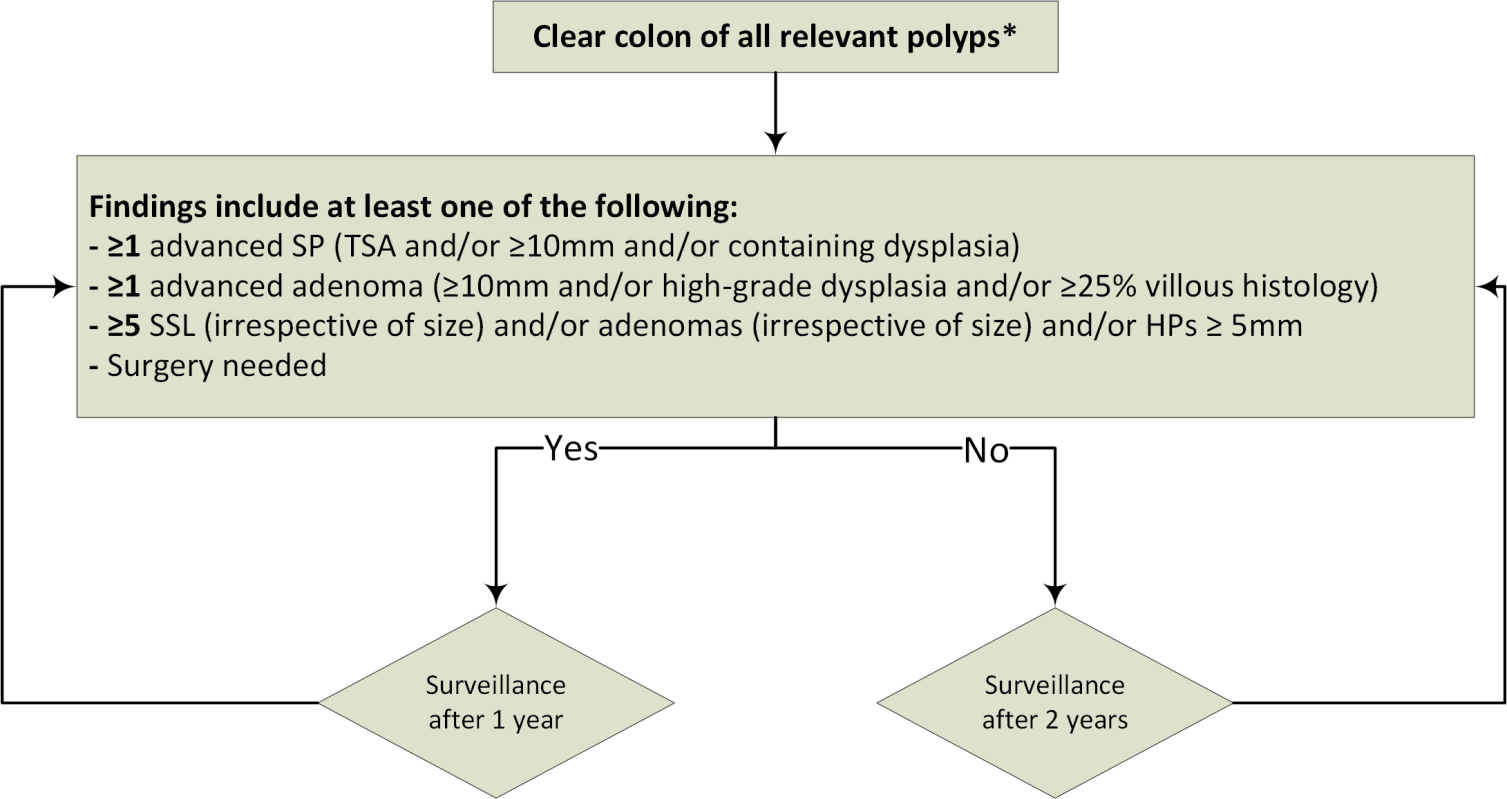
Flow chart of surveillance protocol. *Clear colon of all polyps ≥5 mm, and all polyps <5 mm with the optical aspect of adenoma TSA or SSL. Thus, HPs 1–4 mm may be left in situ. HPs, hyperplastic polyp; SP, serrated polyp; SSL, sessile serrated lesion; TSA, traditional serrated adenoma.

The protocol can be divided into two phases: the clearing phase and the surveillance phase. Surveillance colonoscopies that were scheduled during the study and according to the following protocol will from hereon be referred to as *protocolised surveillance*. All surveillance that took place prior to study inclusion will from hereon be referred to as *non-protocolised surveillance.* A flow chart of the protocol is shown in figure 1.

#### Clearing phase

All patients underwent clearing with complete removal of all polyps ≥5 mm, and all polyps <5 mm with the optical aspect of an adenoma, SSL or TSA (thus, HPs 1–4 mm could be left in situ). If endoscopic clearing was not possible during one procedure, colonoscopies were rescheduled within 6 months until all relevant polyps were removed. A new colonoscopy was also re-scheduled in case the cecum was not intubated and/or when the Boston Bowel Preparation Score was below 6. Patients that had been cleared before the start of this study could be included as well, in case the last recorded clearing colonoscopy was performed according to the above-mentioned quality criteria.

#### Surveillance phase

During surveillance, all polyps ≥5 mm and all polyps <5 mm with the optical aspect of an adenoma or SSLs were removed (HPs 1–4 mm could be left in situ). Each surveillance colonoscopy was scheduled with an interval of either 1 or 2 years, based on the findings at the previous endoscopy (figure 1). Thus, the recommended surveillance interval for each patient could vary based on the findings of each subsequent surveillance colonoscopy (figure 1). In case of multiple colonoscopies in the same surveillance phase (eg, because of poor bowel preparation, incomplete colonoscopy or multiple colonoscopies due to polyp burden), detected lesions from all colonoscopies were accumulated to determine the subsequent surveillance interval.

Patients were recommended a surveillance interval of 1 year in case one or more advanced SPs or adenomas were removed, if cumulatively ≥5 relevant polyps were removed (SSL [irrespective of size] and/or adenomas [irrespective of size] and/or HPs ≥5 mm), or if surgery was needed during the last surveillance/clearing phase. In all other cases, a 2-year surveillance interval was recommended.

The criteria used in this protocol were chosen based on the consensus of the authors, since no risk factors had been established at the time this protocol was drafted in 2012.

### Protocol violation

Protocol violation was defined as a deviation from the required surveillance interval of ≥365 days. In case of violation of the surveillance protocol, only the data prior to the protocol violation were used for our analyses, and the patient was censored at the moment the surveillance colonoscopy should have taken place.

### Colonoscopy quality

All procedures were performed using standard or high-definition white light endoscopy at dedicated endoscopy programmes. Advanced imaging techniques (eg, [virtual] chromoendoscopy and Endocuff) were not routinely used, but could be used based on the preference of the endoscopist. Bowel preparation was performed according to local practice.

### Study endpoints and statistical analyses

Primary endpoint was the cumulative 5-year incidence of CRC alone as well as of advanced neoplasia (AN) (CRC, advanced SP or advanced adenoma), calculated using Kaplan-Meier analyses. All risk factors identified in previous studies (smoking, WHO criteria, previously diagnosed with SPs proximal to the splenic flexure, SPs with dysplasia, advanced SPs, advanced adenomas, synchronous large SP and advanced adenoma, and age at SPS diagnosis), as well as history of CRC, were assessed in univariate and multivariable (adjusted for age, gender and smoking status) Cox regression analysis and were presented as HR with 95% CI.^1–3 20^ A multivariate model was used to perform multiple imputations to adjust for missing variables.

Secondary outcomes were the frequency of 1-year and 2-year surveillance recommendation and the incidence of conversion to colorectal surgery during the surveillance phase. The risk of AN after a 1-year recommendation was compared with the risk of AN after a 2-year recommendation using logistical regression analysis, expressed as OR. Survival curves were produced using RStudio V.1.1.453 (Integrated Development for R. RStudio, Inc., Boston, MA, USA) with survminer package version V.0.4.3. All other analyses were performed using SPSS V.24.

## Results

### Study flow and patient characteristics

**Figure 2:**
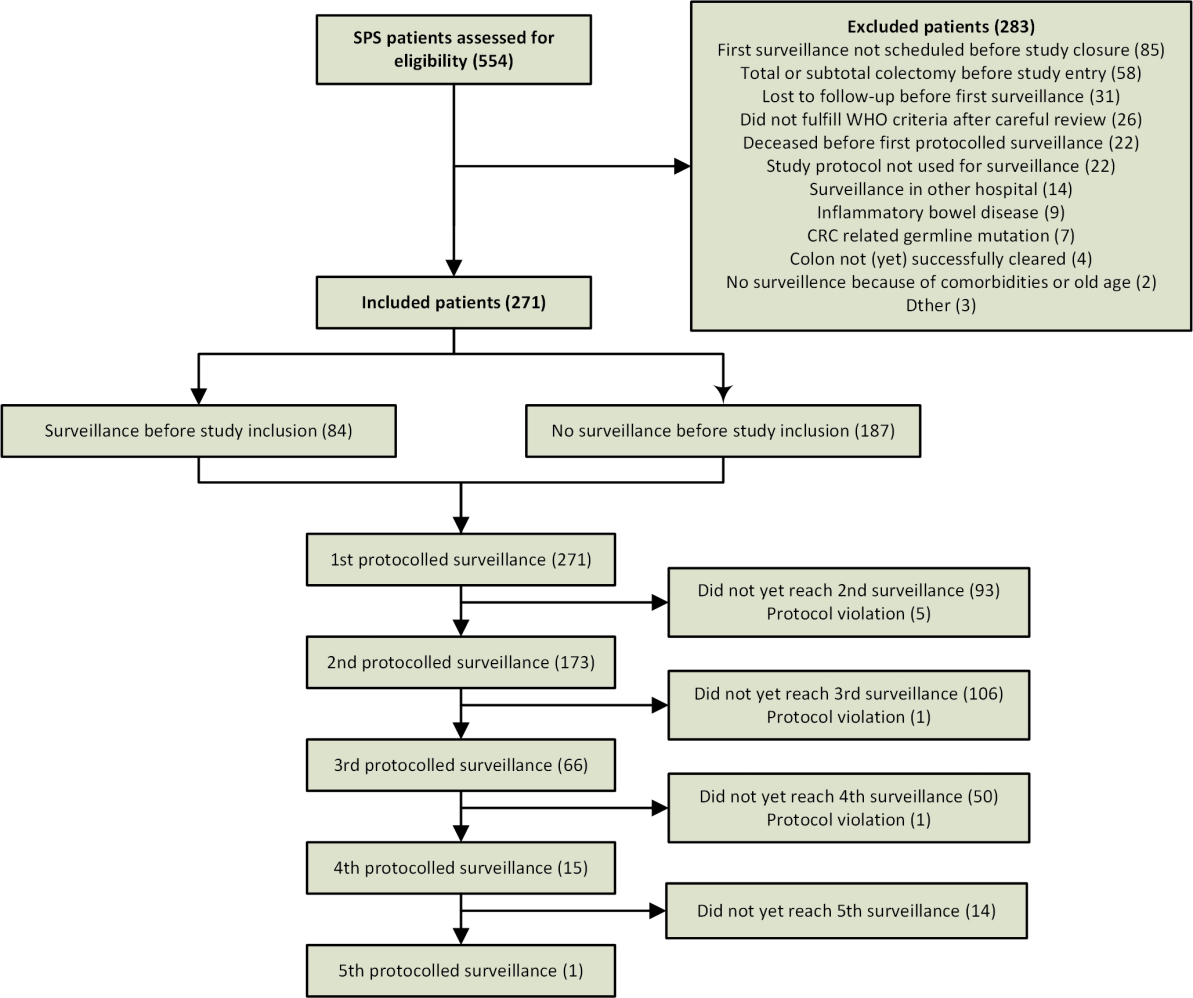
Flow chart of study inclusions. CRC, colorectal cancer; SPS, serrated polyposis syndrome.

Between January 2013 and April 2018, 554 SPS patients were identified in patient registries of the participating centres, and were assessed for eligibility (figure 2). After review, 141 patients did not meet our inclusion and exclusion criteria and were, thus, excluded. Eighty-five patients had to be excluded because they were enrolled too late and thus, their first surveillance was scheduled after study closure. Furthermore, patients were excluded because they did not receive any colonoscopy within the study timeframe (31), because of systematic protocol violation (22) or because their colon was not yet completely cleared (4).

The 271 included patients were followed for a median of 3.6 years (IQR 2.3–4.9 years). Mean age at the start of protocolised surveillance was 60 years (±10), and 130 (48%) were male (table 1). At study inclusion, 99 patients (36.5%) fulfilled WHO criterion I; 99 (36.5%) fulfilled criterion III and 73 (27%) fulfilled criterion I and III. Prior to protocolised surveillance, 67 patients (25%) were diagnosed with CRC, of whom nine had synchronous CRC, six had metachronous CRC and one had both synchronous and metachronous CRC.

**Table 1:** Baseline characteristics for the 271 included patients

Age at diagnosis SPS, mean (SD)	60 (10)
Age at start of prospective follow-up, mean (SD)	62 (9)
Male, n (%)	130 (48%)
Reason first colonoscopy, n (%)	
FOBT-based screening	79 (29%)
Primary colonoscopy screening	14 (5.2%)
Familial CRC risk	36 (13%)
Symptoms	110 (41%)
Pain/discomfort abdomen	27/110 (25%)
Rectal blood loss	29/110 (26%)
Altered defecation pattern	29/110 (26%)
Anaemia	6/110 (5.5%)
Unexplained weight loss	2/110 (1.8%)
Other/unknown	32 (12%)
Family history	
≥1 FDR with CRC, n (%*)	85/250 (34%)*
≥1 FDR with SPS (WHO 1 and/or 3), n (%*)	11/256 (4.3%)*
WHO SPS classification at inclusion, n (%)	
I	99 (36.5%)
III	99 (36.5%)
I and III	73 (27%)
CRC prior to protocolised surveillance phase, n (%)	67 (25%)
Age at diagnosis, median (range)†	61 (19–79)
Multiple CRC, n (%)	16 (24%)
Synchronous	9/16 (56%)
Metachronous	6/16 (37.5%)
Synchronous and metachronous	1/16 (6.2%)
Moment of CRC diagnosis, n (%)†	
Prior to clearing phase	40/67 (60%)
During clearing phase	27/67 (40%)
During surveillance, but prior to study inclusion	0
Total number of colonoscopies prior to clearing	523
Per patient, median (range)	1 (0–15)
Total number of clearing colonoscopies	543
Per patient, median (range)	2 (1–9)
Total no. of surveillance colonoscopies prior to inclusion	202
Per patient, median (range)	0 (0–7)
Total number of prospective protocolised surveillance colonoscopies	570
Per patient, median (range)	2 (1–5)
Total years of prospective follow-up	942 patient-years
Per patient, median (IQR)	3.6 (2.3–4.9)

*Percentage refers to patients for whom variable was available.

†In case patients had multiple (metachronous) CRCs, these values refer to the first CRC.

CRC, colorectal cancer; FDR, first-degree relative; FOBT, faecal occult blood test; SPS, serrated polyposis syndrome.

**Figure 3:**
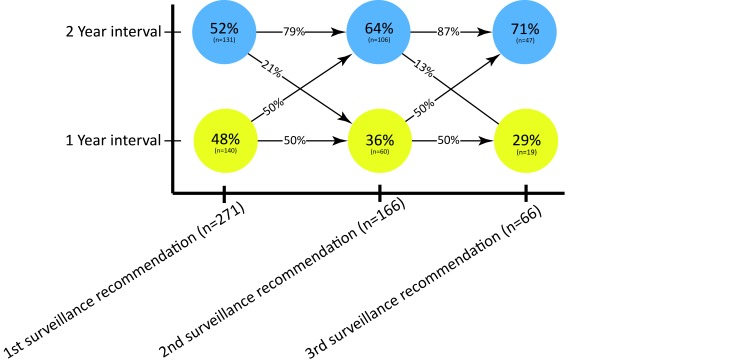
Dynamics of surveillance recommendation 1 year versus 2 years in surveillance round 1–3. Circles represent the proportion of patients in surveillance round 1, 2 and 3 that received a 1-year or 2-year surveillance recommendation. The arrows show the proportion of patients that are recommended the same (horizontal arrows) or a different (diagonal arrows) surveillance interval during subsequent round of surveillance. For example, 79% of the patients recommended a 2-year interval in round 1 are also recommended a 2-year interval in round 2.

Of the 271 included patients, 84 (31%) had already undergone surveillance before inclusion while the remaining 69% underwent their first surveillance endoscopy during follow-up. The majority of patients received a 2-year surveillance recommendation, which increased from 52% after the first surveillance colonoscopy to 71% after the third surveillance colonoscopy (figure 3). A 2-year interval in the first and second surveillance round was followed by another 2-year interval in 79% and 87% of patients, respectively.

All patients underwent at least one protocolised surveillance colonoscopy, which was followed by a second, third, fourth and fifth surveillance colonoscopy in 173, 66, 15 and 1 patients, respectively. Protocol violation led to early dropout in seven patients. A detailed overview of relevant findings during surveillance colonoscopies is presented in table 2.

**Table 2:** Prevalence and number of polyps removed prior to or during clearing, during surveillance prior to inclusion and during protocolised surveillance

	During/prior to clearing (n=271)	Surveillance prior to study inclusion (n=84)	Surveillance after study inclusion using personalised surveillance protocol				
			Surveillance 1 (n=271)	Surveillance 2 (n=173)	Surveillance 3 (n=66)	Surveillance 4 (n=15)	Surveillance 5 (n=1)*
Patients with CRC, n (%)	69 (25%)	0	0	1	1	0	0
Patients with ≥1 polyp	271 (100%)	81	214 (79%)	132 (76%)	56 (85%)	15 (100%)	1
Number of polyps per patient, median (range)	22 (1–157)	8 (0–39)	3 (0–19)	2 (0–20)	2 (0–13)	3 (1–9)	2
Patients with ≥1 advanced polyp†	237 (87%)	25 (30%)	73 (27%)	31 (18%)	12 (8%)	6 (40%)	0
Patients with at least one:							
Any SP	271 (100%)	78 (93%)	195 (72%)	121 (70%)	45 (68%)	13 (87%)	1
HP	249 (92%)	77 (92%)	148 (55%)	83 (48%)	25 (38%)	11 (73%)	0
SSL	215 (79%)	37 (44%)	106 (39%)	64 (37%)	27 (41%)	4 (27%)	0
TSA	15 (5.9%)	4 (5%)	1 (0.4%)	2 (1.2%)	1 (1.5%)	0	0
SP ≥10 mm	192 (71%)	13 (16%)	58 (21%)	28 (16%)	7 (11%)	4 (27%)	0
SP with dysplasia	61 (23%)	7 (8%)	7 (3%)	5 (2.9%)	5 (8%)	0	0
Median number of SP per patient (range):							
Any SP	17 (1–116)	6 (0–31)	2 (0–17)	2 (0–20)	1.5 (0–13)	2 (0–8)	2
HP	9 (0–114)	4 (0–31)	1 (0–13)	0 (0–10)	0 (0–8)	1 (0–7)	0
SSL	4 (0–55)	0 (0–23)	0 (0–15)	0 (0–11)	0 (0–13)	0 (0–3)	0
TSA	0 (0–7)	0 (0–5)	0 (0–6)	0 (0–1)	0 (0–1)	0 (0–0)	0
SP ≥10 mm	2 (0–25)	0 (0–5)	0 (0–7)	0 (0–4)	0 (0–3)	0 (0–2)	0
SP with dysplasia	0 (0–14)	0 (0–5)	0 (0–6)	0 (0–2)	0 (0–3)	0 (0–0)	0
Patients with at least one adenoma:	216 (80%)	58 (69%)	113 (42%)	60 (35%)	21 (32%)	7 (47%)	0
Advanced adenoma ‡	131 (48%)	8 (9%)	20 (7.4%)	3 (2.3%)	1 (1.5%)	2 (13%)	0
Median number of adenomas per patient	3 (0–41)	1.5 (0–16)	0 (0–10)	0 (0–8)	0 (0–8)	0 (0–4)	0
Advanced adenoma	0 (0–7)	0 (0–1)	0 (0–2)	0 (0–2)	0 (0–1)	0 (0–1)	0

*This column represents only one patient. Therefore, no median or range is given.

†Advanced polyp: advanced adenoma (≥25% villous histology, high-grade dysplasia and ≥10 mm in diameter)/advance.

‡Advanced adenoma: ≥25% villous histology, high-grade dysplasia and ≥10 mm in diameter.

CRC, colorectal cancer; HP, hyperplastic polyp; FDR, first-degree relative; FOBT, faecal occult blood test; SP, serrated polyp; SPS, serrated polyposis syndrome; SSL, sessile serrated lesion; TSA, traditional serrated adenoma.

### CRC under protocolised surveillance

**Figure 4:**
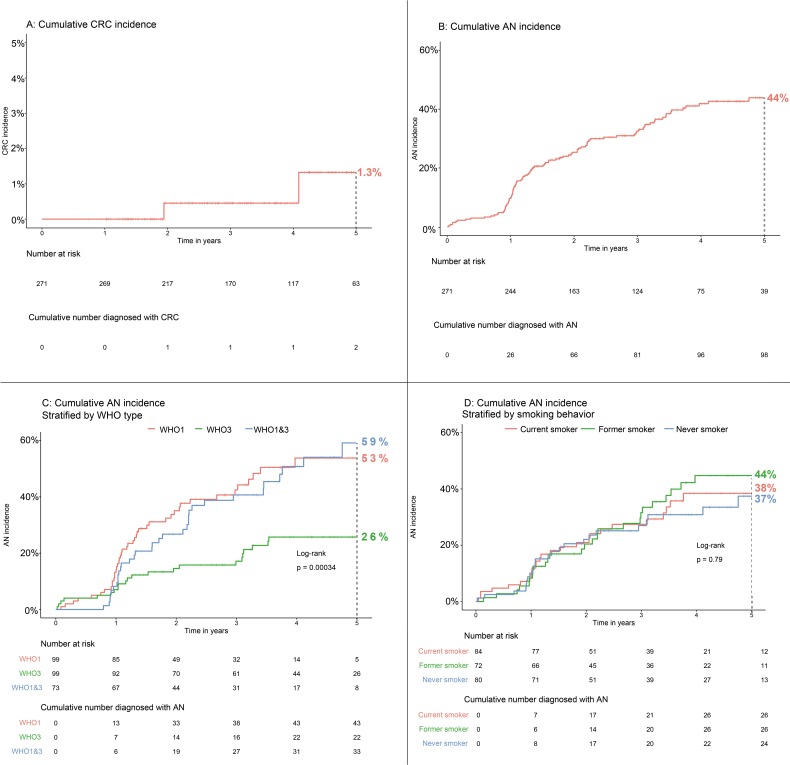
Cumulative 5-year incidence of CRC (A) and advanced neoplasia (B–D). The p value displayed in panel C and D was calculated using a log-rank test, comparing all WHO subtypes (panel C) and smoking behaviour (panel D). AN, advanced neoplasia (defined as CRC, advanced adenoma or advanced SP); CRC, colorectal cancer; SP, serrated polyp.

Two patients were diagnosed with CRC while under protocolised endoscopic surveillance, corresponding to a cumulative 5-year CRC incidence of 1.3% (95% CI 0% to 3.2%, figure 4A). The CRCs occurred in surveillance phase 2 and 3, respectively. Detailed characteristics are presented in online supplementary table 1. Briefly, the first CRC occurred 1 year after piecemeal removal of a tubulovillous adenoma with HGD, and was preceded by a scar inspection 6 months earlier during which the scar could not be located. The second CRC occurred after a 2-year recommendation. It protruded from inside an anastomosis of an earlier right-sided hemicolectomy, and histologically and endoscopically appeared to be a local recurrence of a CRC that had been resected 6 years earlier.

10.1136/gutjnl-2018-318134.supp1 Supplementary data

### AN under protocolised surveillance

The cumulative 5-year incidence of AN during follow-up was 44% (95% CI 37% to 51%, figure 4B). The risk of developing AN during follow-up was strongly associated with WHO subtype: 53% (95% CI 39% to 65%), 26% (95% CI 16% to 35%) and 59% (95% CI 40% to 72%) for WHO I, III, and I and III, respectively. Compared with patients diagnosed with WHO I, patients fulfilling WHO III had a HR of 0.38 (95% CI 0.22 to 0.63, p<0.001) for developing AN. Adjusted for age at inclusion, smoking status and gender, the HR remained unchanged (table 3 and figure 4C). Patients fulfilling only WHO criterion I had the same risk as patients diagnosed with both WHO criteria I and III (HR 0.91, 95% CI 0.58 to 1.43, p=0.68).

**Table 3:** Risk factors for time to advanced neoplasia during follow-up

	Advanced neoplasia during surveillance		Univariate HR (95% CI)	P value	Multivariable HR*(95% CI)	P value
	No	Yes				
Gender, n (%)						
Male	83 (64%)	47 (36%)	1		1	
Female	90 (64%)	51 (36%)	0.95 (0.64 to 1.41)	0.81	0.99 (0.66 to 1.50)	0.97
Smoking status						
Never smoker	56 (70%)	24 (30%)	1		1	
Former smoker	46 (65%)	25 (35%)	1.22 (0.71 to 2.08)	0.48	1.20 (0.68 to 2.13)	0.52
Current smoker	58 (69%)	26 (31%)	1.10 (0.62 to 1.95)	0.75	1.09 (0.61 to 1.95)	0.76
WHO criterion at study inclusion†						
WHO I	56 (57%)	43 (43%)	1		1	
WHO III	77 (78%)	22 (22%)	0.38 (0.22 to 0.63)	<0.001	0.38 (0.22 to 0.64)	<0.001
WHO I and III	40 (55%)	33 (45%)	0.91 (0.58 to 1.43)	0.68	0.89 (0.56 to 1.40)	0.6
Age at SPS diagnosis			0.98 (0.96 to 1.01)	0.15	0.99 (0.96 to 1.01)	0.16
≥1 advanced adenomas‡						
No	93 (66%)	47 (34%)	1		1	
Yes	80 (61%)	51 (39%)	1.21 (0.82 to 1.80)	0.34	1.24 (0.82 to 1.87)	0.31
≥1 advanced SP‡						
No	45 (69%)	20 (31%)	1		1	
Yes	128 (62%)	78 (38%)	1.46 (0.89 to 2.39)	0.13	1.46 (0.89 to 2.40)	0.13
≥1 SP proximal to splenic flexure†						
No	27 (59%)	19 (41%)	1		1	
Yes	146 (65%)	79 (35%)	0.84 (0.51 to 1.38)	0.49	0.86 (0.51 to 1.43)	0.95
≥1 SP with dysplasia‡						
No	131 (62%)	79 (38%)	1		1	
Yes	42 (69%)	19 (31%)	0.82 (0.50 to 1.36)	0.44	0.84 (0.51 to 1.39)	0.5
≥1 SP≥10 mm and ≥1 advanced adenoma‡						
No	167 (65%)	91 (35%)	1		1	
Yes	6 (46%)	7 (54%)	1.270 (0.59 to 2.74)	0.54	1.21 (0.56 to 2.64)	0.63
CRC‡						
No	131 (64%)	73 (36%)	1		1	
Yes	42 (63%)	25 (37%)	1.14 (0.74 to 1.80)	0.56	0.80 to 2.07	0.31

*Adjusted for potential confounders: age, gender and smoking status.

†WHO criterion I: ≥5 SP proximal to sigmoid colon with at least two being ≥10 mm in diameter; WHO criterion III: ≥20 SP of any size, spread throughout the colon.^5^

‡Diagnosed prior to or during clearing phase.

CRC, colorectal cancer; SP, serrated polyp; SPS, serrated polyposis syndrome.

None of the other previously identified CRC risk factors (gender, smoking status, advanced adenoma, SP with dysplasia, advanced SP, large SP with synchronous advanced adenoma, SP proximal to the splenic flexure or previous history of CRC) were associated with the risk of developing AN during surveillance in univariate and multivariable analyses (table 3 and figure 4D).

Because patients that received surveillance prior to study inclusion might have a different risk of AN than those that received their first surveillance during our study, we performed additional sensitivity analyses. These showed that patients that had received one or more surveillance colonoscopies prior to study inclusion had a slightly lower risk of developing AN during our study than patients that received their first surveillance during our study (HR 0.64, 95% CI 0.41 to 0.99; p=0.047)

### AN after a 1-year versus 2-year surveillance interval

In 255 occasions, a surveillance recommendation according to our protocol was followed by a subsequent surveillance endoscopy. AN was detected in 31 of 127 (24.4%) colonoscopies that were performed after a 1-year surveillance recommendation, compared with 20 of 128 (15.6%) colonoscopies performed after a 2-year surveillance recommendation (OR 0.57, 95% CI 0.31 to 1.07, p=0.08). Incidence of other polyp subtypes after 1-year versus 2-year recommendation is displayed in online supplementary table 2.

10.1136/gutjnl-2018-318134.supp2 Supplementary data

### Surgery

During protocolised surveillance, surgery was required in three patients. The first patient underwent laparotomy and adhesiolysis because of bowel obstruction due to previous surgery. The other two patients underwent a proctocolectomy and subtotal colectomy, respectively, both because of CRC. Both had undergone a right-sided hemicolectomy for CRC in the past. No surgery was performed for the indication polyp burden or a single endoscopically unresectable polyp.

## Discussion

In this large prospective cohort study, we assessed the safety and effectivity of an individualised surveillance protocol for patients diagnosed with SPS. Our protocol showed to be effective in decreasing colonoscopy burden while at the same time achieving low incidence of CRC. During a median follow-up of 3.6 years, the cumulative 5-year incidence of CRC was 1.3%, which is similar to the incidence reported in recent studies,^1 6 21^ and lower than incidence rates in two retrospective cohort studies.^3 4^ In comparison, this incidence rate is identical to that of a regular postpolypectomy population after resection of intermediate-risk adenomas.^22^ The cumulative 5-year incidence of AN, on the other hand, was high (44%) and strongly associated with WHO subtype. Patients diagnosed with WHO criterion I or I and III were more than twice as likely to develop AN during surveillance compared with patients diagnosed with WHO criterion III only. The use of our protocol seems safe and effective with little need for surgical intervention. After three consecutive surveillance colonoscopies, two-thirds of the patients were appointed 2-year surveillance intervals according to the protocol. The extended 2-year interval did not result in an increased incidence of AN. Indeed, a non-significant trend was seen for a lower risk of AN after a 2-year recommendation (OR 0.57, p=0.08)

The high AN incidence confirms that close endoscopic surveillance of SPS patients is warranted.^3^ However, we believe (more) stringent surveillance than the regimen described by our surveillance protocol will not further reduce CRC incidence. After all, one of the two CRCs that occurred during our study occurred after a surveillance recommendation of 1 year. The other CRC, occurring after a 2-year recommendation, was a local recurrence protruding from inside the anastomosis. Although more stringent surveillance could have led to earlier detection of this recurrence, it would not have been prevented. Since none of the CRCs in our cohort can, thus, be attributed to the lengthened surveillance intervals, extension of the surveillance interval to 2 years based on our protocol seems safe and feasible. The US guidelines recommend annual surveillance,^12^ and although European guidelines generally recommend surveillance every 1–2 years, reported median surveillance intervals range from 1.1 to 1.3 years.^1–3^ As our protocol predominantly led to a 2-year surveillance recommendation, a substantial reduction in colonoscopy burden could be achieved compared with current practice. As a comparison, in case annual surveillance would have been provided in our cohort, about 942 colonoscopies would have been performed during the 942 patient-years of prospective follow-up. Using our protocol, however, only 570 colonoscopies were performed, which means about 372 (39%) fewer colonoscopies were performed using our protocol compared with annual surveillance. Further improvement of risk stratification should aim to further reduce colonoscopy burden without increasing AN risk. However, no ‘acceptable’ incidence of AN per surveillance colonoscopy has been established for SPS patients. Although our study might provide benchmarks in this regard, the incidence rate that should trigger early surveillance can be debated. Some might argue that surveillance intervals can be extended up until a point where SPS patients have an AN incidence of 30% or even 40%, whereas others would rather shorten surveillance intervals until AN incidence would be below 15%. In our study, we found an AN incidence of 24.4% following a 1-year interval and 15.6% after a 2-year interval. Comparably, a recent large retrospective cohort study reported an average AN incidence of 24% per surveillance colonoscopy.^3^ Taken together, this suggests AN incidence rates of 20%–25% per surveillance colonoscopy might be inevitable.

To further improve risk stratification, we assessed risk factors that have previously been identified (smoking status, age at inclusion, fulfilment of WHO criterion I and III, previous diagnosis of an advanced adenoma, advanced SP, synchronous large SP and advanced adenoma, SP proximal to the splenic flexure or an SP with dysplasia).^1–3 20^ Fulfilment of WHO criterion III was inversely associated with a patient’s risk of AN during follow-up. This association was remarkably strong, and might suggest that surveillance intervals can be extended for patients that solely fulfil WHO criterion III. None of the other risk factors was associated with AN during surveillance in our cohort. This could be explained by the fact that the studies that initially identified these risk factors assessed their association with a history of CRC. Almost all CRCs included in those analyses occurred *prior to surveillance*. In contrast, in our study, we assessed the association between these risk factors and the risk of AN *during surveillance*. The incongruence between our and previous findings suggests that risk factors associated with CRC before surveillance are not necessarily applicable for the risk of developing AN during surveillance. However, although our cohort is large compared with other studies on SPS, it is still relatively small, and perhaps was not powered to detect subtle correlations between the assessed risk factors and AN.

We believe our study has several strengths. First of all, we describe the largest prospective cohort of SPS patients to date, and this study was the first to assess a personalised surveillance protocol for SPS patients, allowing risk stratification for a mixed population with a variable CRC risk. As surveillance guidelines are currently based on low-quality evidence, many authors have expressed the urge of such prospective evaluation of surveillance.^1–3 12^ Our study now provides an evidence-based surveillance strategy for SPS patients, which could be of help to establish future surveillance guidelines. The international multicenter design, as well as the fact that both academic and non-academic centres were included, warrants a good external validity. Nonetheless, several limitations have to be acknowledged as well. First, our median follow-up duration of 3.6 years might be considered short to monitor CRC occurrence. Longer follow-up with more rounds of surveillance is needed to assess the long-term CRC incidence. Second, with only two cases of CRC during surveillance, we were not able to assess risk factors for the development of CRC during surveillance because the sample size required for such analyses would be a multitude of our study population. Nonetheless, we were able to evaluate risk factors for AN during follow-up, which is likely to be strongly associated with one’s risk of developing CRC. Third, some patients received only one protocolised surveillance colonoscopy, whereas others received five. The more colonoscopies a patient underwent during the 5-year study period, the more likely it is that he or she received several 1-year surveillance recommendations instead of 2-year surveillance recommendations, owing to advanced polyps or a high number of non-advanced polyps. Therefore, the patients that underwent colonoscopies in surveillance phase 3, 4 or 5 are predominantly high-risk patients that have a tendency to harbour advanced findings. The described prevalence of (advanced) polyps in surveillance phase 3–5 (table 2) might, therefore, be inflated due to confounder by indication. However, because our survival analyses were not influenced by the number of surveillance colonoscopies, our main outcome measures were not affected by such bias. Finally, some of the patients enrolled in our study (31%) had already received one or more surveillance colonoscopies prior to study inclusion. This is relevant, since AN incidence seemed to decrease after several rounds of surveillance, which can be seen in table 2 and figure 3B. Patients that already underwent surveillance prior to study inclusion could, therefore, be expected to have a lower AN incidence than patients that underwent their first surveillance as part of the study. This effect was confirmed in a sensitivity analysis: patients with surveillance prior to study inclusion were at lower risk of AN than patients without surveillance prior to study inclusion (HR 0.64, 95% CI 0.41 to 0.99, p=0.047). Since the majority (69%) of our cohort did not undergo surveillance prior to inclusion, we expect this effect has not substantially influenced our results. Nonetheless, a slightly higher AN incidence than reported here might be expected in a population without previous surveillance.

All in all, our results suggest that the proposed surveillance protocol can safely be implemented for SPS patients without risking an increase in CRC incidence. Future studies should aim to reduce the colonoscopy burden in true low-risk patients even further. Since a 2-year recommendation was not associated with increased AN incidence, perhaps further extension beyond 2 years might be safe for these patients as well. Our results, furthermore, indicate that patients that only fulfil WHO criterion III might be a group of low-risk, and possibly a 3-year or even a 5-year surveillance interval might be safe for this group. However, it is important to note that SPS patients are a group of high-risk patients, and an extension of surveillance intervals from the currently studied protocol should be done with caution and be part of prospective studies in expert centres.

In conclusion, in this largest prospective cohort of SPS patients to date, we present and assessed a new personalised surveillance strategy for the treatment and surveillance of this prevalent disease. While achieving a substantial reduction of colonoscopy burden, CRC incidence in our cohort was similar to most previously reported incidence rates. Our protocol will likely lead to a substantial reduction in colonoscopy burden. Future efforts will be made to further reduce colonoscopy burden, especially for patients only fulfilling WHO criterion III.
