# Correction: *Personalised surveillance for serrated polyposis syndrome: results from a prospective 5-year international cohort study*

**DOI:** 10.1136/gutjnl-2018-318134corr1

**Published:** 2020-09-03

Bleijenberg AG, IJspeert JE, van Herwaarden YJ, *et al*. Personalised surveillance for serrated polyposis syndrome: results from a prospective 5-year international cohort study. *Gut* 2020;69:112–21. doi:10.1136/gutjnl-2018-318134

The author name Iris D Nagetaal should be Iris D Nagtegaal.

