# Supplementary material for: Personalised surveillance for serrated polyposis syndrome: results from a prospective 5-year international cohort study
Source: Gut. 2019 Apr 13;69(1):112–21. doi: 10.1136/gutjnl-2018-318134 (PMC6943249; doi:10.1136/gutjnl-2018-318134)
Supplement: Supplementary data [file gutjnl-2018-318134supp001.pdf]

Supplementary table 1: Patient characteristics of patients with CRC during protocolized surveillance

	**Patient 1**	**Patient 2**
**Moment of CRC**	2nd protocolized surveillance colonoscopy	3rd protocolized surveillance colonoscopy
**Relevant medical history**	1998: Cervical cancer, treated with chemotherapy & radiation<br>2011: T4N0M0 cecum CRC. Treated with rightsided hemicolectomy.<br>2014:Piecemeal resection of tubulovillous adenoma with high-grade dysplasia from transverse colon.	2011: T3N0M0 CRC in transverse colon, treated with right-sided hemicolectomy
**Year of diagnosis**	2015	2017
**Recommended surveillance interval after previous colonoscopy according to protocol**	1 year	2 years
**Patient characteristics**		
Age at SPS diagnosis	53	57
Age at CRC diagnosis	55	63
Gender	Female	Female
WHO criteria for SPS diagnosis	WHO I	WHO I&III
Number of surveillance colonoscopies until diagnosis	0	0
**Time since clearing (months)**	23 months	49 months
**Time since last colonoscopy (months)**	7 months	25 months
**Neo-terminal ileum intubated**		Yes
**BBPS ≥2 in each segment during preceding colonoscopy?**	Yes	Yes
**Tumour features**		
Tumour staging (TNM)	T3N0M0	T3N1M0
Location	Transverse colon	At the anastomosis of a previous hemicolectomy
**Management**	Proctocolectomy	Subtotal colectomy

CRC, colorectal cancer; SPS, serrated polyposis syndrome; WHO, world health organization; BBPS, Boston bowel preparation scale
