# Supplementary material for: Personalised surveillance for serrated polyposis syndrome: results from a prospective 5-year international cohort study
Source: Gut. 2019 Apr 13;69(1):112–21. doi: 10.1136/gutjnl-2018-318134 (PMC6943249; doi:10.1136/gutjnl-2018-318134)
Supplement: Supplementary data [file gutjnl-2018-318134supp002.pdf]

Supplementary table 2: Findings following 1- and 2-year surveillance recommendations

	Preceding surveillance recommendation					
	1 year (n=127)		2 years (n=128)			
	Yes	No	Yes	No	OR (95%CI)	P
**High polyp burden*, n(%)**	52 (41%)	75 (59%)	24 (19%)	104 (81%)	0.33 (0.19-0.59)	<.001
**≥1 Advanced neoplasia**	31 (24%)	96 (76%)	20 (16%)	108(84%)	0.57 (0.31-1.07)	.082
**≥1 Conventional adenoma**	45 (35%)	82 (65%)	43 (34%)	85 (66%)	0.92 (0.55-1.55)	.76
**≥1 Advanced adenoma**	4 (3.2%)	123 (97%)	9 (7.0%)	119 (93%)	2.33 (0.70-7.76)	.17
**≥1 Sessile serrated lesion**	55 (43%)	72 (57%)	44 (34%)	84 (66%)	0.69 (0.41-1.14)	.14
**≥1 Serrated polyp ≥10mm**	25(20%)	102 (80%)	14 (11%)	114 (89%)	0.50 (0.25-1.02)	.06
**≥1 Serrated polyp with dysplasia**	4 (3.2%)	123 (97%)	1 (0.78%)	127 (99%)	0.24 (0.03-2.20)	.21

* High polyp burden: Advanced neoplasia and/or ≥5 non-advanced relevant polyps (HP ≥5mm, SSLs or tubular adenomas)

Abbreviations: OR, odds ratio; AN, advanced neoplasia; HP, hyperplastic polyp; SSL, sessile serrated lesion;
